# Scattering of MCF7 Cells by Heregulin ß-1 Depends on the MEK and p38 MAP Kinase Pathway

**DOI:** 10.1371/journal.pone.0053298

**Published:** 2013-01-07

**Authors:** Rintaro Okoshi, Chung-Li Shu, Sayoko Ihara, Yasuhisa Fukui

**Affiliations:** 1 Institute of Cellular and System Medicine, National Health Research Institutes, Zhunan, Taiwan, Republic of China; 2 Division of Applied Biological Chemistry, Graduate School of Agricultural and Life Sciences, University of Tokyo, Tokyo, Japan; 3 Laboratory of Signal Transduction, Hoshi University, Tokyo, Japan; West Virginia University, United States of America

## Abstract

Heregulin (HRG) β1 signaling promotes scattering of MCF7 cells by inducing breakdown of adherens and tight junctions. Here, we show that stimulation with HRG-β1 causes the F-actin backbone of junctions to destabilize prior to the loss of adherent proteins and scattering of the cells. The adherent proteins dissociate and translocate from cell–cell junctions to the cytosol. Moreover, using inhibitors we show that the MEK1 pathway is required for the disappearance of F-actin from junctions and p38 MAP kinase activity is essential for scattering of the cells. Upon treatment with a p38 MAP kinase inhibitor, adherens junction complexes immediately reassemble, most likely in the cytoplasm, and move to the plasma membrane in cells dissociated by HRG-β1 stimulation. Subsequently, tight junction complexes form, most likely in the cytoplasm, and move to the plasma membrane. Thus, the p38 MAP kinase inhibitor causes a re-aggregation of scattered cells, even in the presence of HRG-β1. These results suggest that p38 MAP kinase signaling to adherens junction proteins regulates cell aggregation, providing a novel understanding of the regulation of cell–cell adhesion.

## Introduction

Under certain conditions *in vitro*, some aggregated epithelial cells can lose their cell–cell interaction, acquire higher cell motility and dissociate. Although the mechanism for epithelial cell dissociation (also known as scattering) is not clear, it likely reflects some response to unknown stimuli *in vivo.*

Epithelial cells form adherens and tight junctions to connect with neighboring cells. Through this connection, cells collaborate during various processes [1]–[8]. When cells are trypsinized and plated on dishes, they move around until they contact other cells. Nectins present on the surface of each cell interact to form the initial connection between two cells [9], [10]. After this, E-cadherin, occludin, and claudin proteins of neighboring cells bind and produce adherens and tight junctions [11]–. These interactions appear to be programmed to form complexes.

Adherens junctions and tight junctions of epithelial cells work together to play many important roles in the cell. It has been suggested that both junction complexes associate with the actin cytoskeleton [2], [13]. The transmembrane protein of adherens junctions, E-cadherin, interacts with intracellular proteins, including β-catenin, p120-catenin, and α-catenin [7]. E-cadherin is an important marker for cell recognition between neighboring cells. Tight junctions contain the transmembrane proteins occludin and claudin, which interact with ZO-1, 2, or 3 [5].

Dissociation of epithelial cells has been widely studied in various cell lines, including MDCK cells, MCF7 cells, and keratinocytes. MDCK cells stimulated by hepatocyte growth factor/dissociate factor (HGF) undergo dissociation [14], [15]. MEK and the phosohatidylinositol 3- kinase (PI3K) pathways have been implicated in the dissociation of MDCK cells [16]–[18]. Rac1 is proposed to negatively regulate disruption of adherens junctions caused by IQGAP in these cells [19]–[23]. In contrast, keratinocytes and other cell lines undergo dissociation by stimulation with EGF. In these cases, prolonged activation of Rac1 is required for dissociation of the cells [24]–[26]. Therefore, keratinocytes may have different dissociation mechanisms from MDCK cells. MCF7 cells are breast cancer cells that possess epithelial cell characteristics, grow as monolayer-aggregates, and form tight and adherens junctions at cell–cell contacts. Additionally, treatment with HRG-β1 causes increased motility and scattering of MCF7 cells [27]. Indeed, HRG-β1 can enhance invasiveness of cancers [28]. Because HRG-β1 signaling is different from HGF and EGF signaling, it is likely that the regulation of dissociation in MCF7 cells is distinct from the cells mentioned above.

The HRG proteins form a family of four members: HRG1, HRG2, HRG3, and HRG4. HRGs are involved in the regulation of cell proliferation, differentiation, migration, apoptosis, and cell survival [29], [30]. They contain unique N-terminal domains, Ig-like domains, and EGF-like domains. In most cases only the EGF-like domains of heregulins can mediate various cellular responses, including cell growth and scattering. ErbB3 or ErbB4 are receptors of HRG-β1. It has been shown that MCF7 cells do not express ErbB4, which suggests that ErbB3 is important for dissociation in these cells. ErbB3 is closely related to tyrosine kinases, but whether it has enzymatic activity is still unclear. ErbB3 likely acts as a substrate for other EGF family receptor tyrosine kinases rather than an enzyme. Therefore, ErbB3 requires a partner to form a complete receptor. Indeed, ErbB3 binds to ErbB2, which has tyrosine kinase activity [31], [32]. The ErbB2/ErbB3 receptor heterodimer activates several signaling pathways, such as the PI3K and MEK cascades. In turn, PI3K regulates Akt and p38 MAP kinase signaling in MCF7 cells [33], [34].

Signaling of ErbB2/ErbB3 is closely related to the formation of cancer. Overexpression of ErbB2, which may activate the ErbB2/ErbB3 pathway, is often detected in breast cancer and associated with malignancy. Perturbing ErbB2 activity with an anti-ErbB2 antibody has been used for breast cancer therapy [35]. Thus, scattering induced by this signaling pathway may help researchers understand the malignancy of ErbB2/ErbB3-dependent tumors. Here, we discuss the mechanisms and signaling related to dissociation and aggregation of cells.

## Materials and Methods

### Reagents Used in this Study

Anti-E-cadherin and anti-β-catenin, antibodies were purchased from BD Biosciences (Franklin Lakes, NJ) and Millipore Co. Ltd. (Billerica, MA). Anti-p120 catenin, anti-phospho-p38 MAP kinase, anti-ERK, and anti-phospho-ERK antibodies were from Cell Signaling Technology Inc. (Danvers, MA). Anti-occludin and Anti-ErbB2 antibodies were from Zymed Laboratory Inc. (South San Francisco, CA). Anti-ErbB3 and anti-p38 MAP kinase antibodies were from Santa Cruz Biotechnology Inc. (Santa Cruz, CA). Anti-Rac1 antibody was from Millipore Co. Ltd. TRITC-conjugated rhodamine-phalloidin was from Sigma-Aldrich Corp. (St. Louis, MO). EGF domain of HRG-β1 was from R&D Systems (Minneapolis, MI). Secondary antibodies conjugated with Alexa 488 or Alexa 594 was from GE Healthcare (London, UK). The p38 MAP kinase inhibitor (SB202190), the MEK1 inhibitor (PD98059), and the MEK1/MEK2 inhibitor (U0126) were from Wako Co. Ltd. (Tokyo, Japan). Anti-ZO-1 was a kind gift from Dr. S. Tsukita.

### Cell Culture

MCF7 cells, a kind gift from Dr. T. Yamori, were cultured in Dulbecco’s modified minimal essential medium supplemented with 5% fetal calf serum.

### Immunofluorescence

Cells were fixed with 10% formaldehyde and permeabilized by PBS containing 0.2% Triton X100. The samples were treated with primary antibody for 1 h and then with secondary antibody for 30 min. The samples were observed under a confocal laser microscope using a 40X UPlanFL N lens with sectioning of 0.25 µm. Images from the section showing the middle of the cell was used in this paper (Olympus FV300, Olympus, Tokyo). Intensity of fluorescence was measured using the quantifying mode of this microscope, which allows intensity of the signal to be measured in a user-defined region.

### Western Blotting, Immunoprecipitation, and Fractionation of the Cells

Western blotting was done using 1% skim milk as a blocking reagent and detected by an ECL system as described before [36]. For immunoprecipitation, cells were lysed in a buffer containing 10 mM Tris-HCl (pH 7.5), 150 mM NaCl, 5 mM EDTA, and 1 mM PMSF. Protein A sepharose bound to appropriate antibodies was added to the lysate. After washing the beads with the lysis buffer three times, the protein bound to the beads was detected by Western blotting.

For fractionation, cells were suspended in a buffer containing 10 mM Tris-HCl (pH 7.5) and 10 mM NaCl with a proteinase inhibitor cocktail, and homogenized with a Dounce homogenizer. After removing the nuclei by low-speed centrifugation, the cytoplasmic fraction was further fractionated to membrane and cytosolic fractions by spinning at 45,000 rpm for 60 min. The supernatant and the precipitate were used as cytosolic and membrane fractions, respectively.

### Time-lapse Imaging

Imaging was performed using a Leica A7 6000 LX time-lapse imager. Cells were cultured in 3.5 cm dishes at appropriate density and applied to the imager. If addition of HRG-βl was required, it was added just before application to the imager. SB202190 was added to the cells without removing the dishes from the imager and without disturbing the operation of the imager. The pictures were taken every 10 min. Representative photos are shown in the figures.

## Results

### HRG β-1 Induces Dissociation of MCF7 Cells

**Figure 1:**
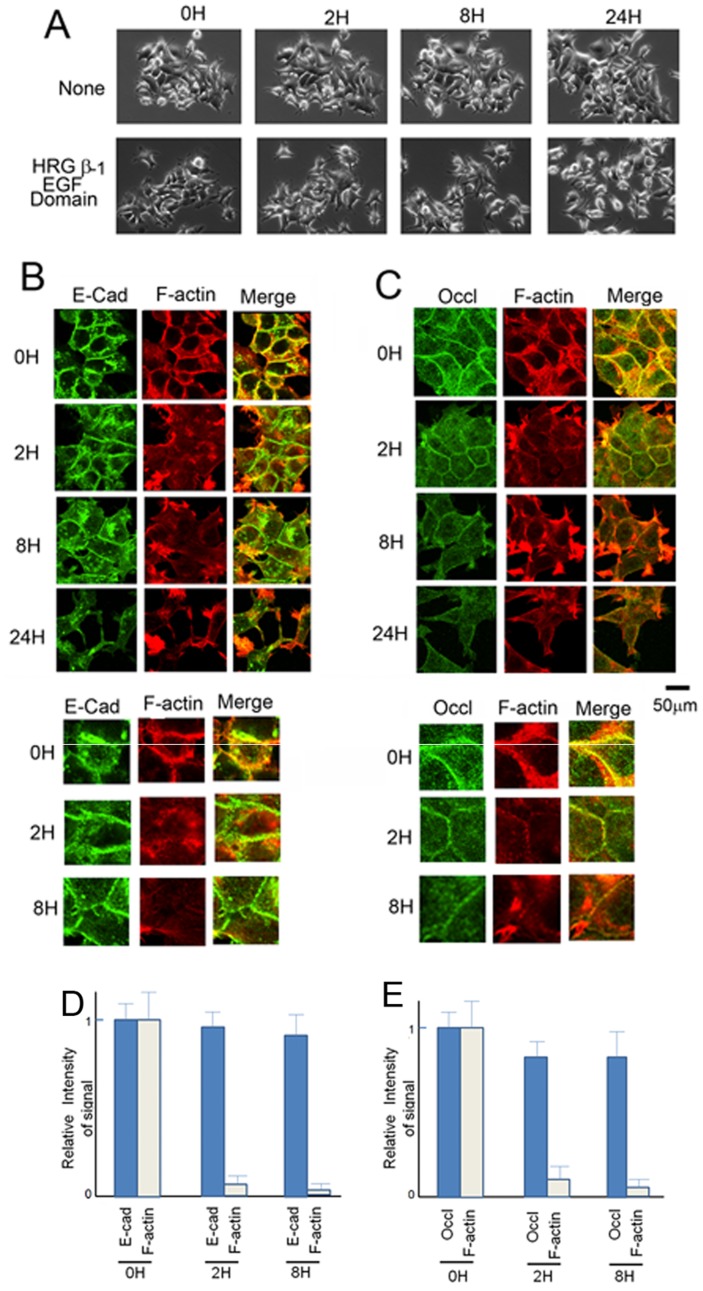
Dissociation of MCF7 cells after HRG treatment. A) Overnight culture of MCF7 cells were treated with or without HRG-β1 (100 ng/ml) for the indicated periods. Time-lapse imaging of the cells was performed, with the relevant times displayed. B) and C) Localization of E-cadherin, occludin, and F-actin after treatment of the cells with HRG-β1. MCF7 cells were treated the EGF domain of HRG-β1 (100 ng/ml) for the indicated time. Cells were stained for E-cadherin B) or occludin C) together with F-actin. 2 h after HRG-β1 treatment, F-actin had almost disappeared from the cell–cell contact, whereas E-cadherin and occludin were readily detectable. Enlarged images of E-cadherin, occludin, and F-actin are shown in the lower part. D) and E), Intensity of the signals of E-cadherin, occludin, and F-actin at the cell-cell contact was measured and expressed in the graph. Intensity of the control samples were expressed by 1.0. The error bars show standard deviation.

MCF7 cells were treated with the EGF domain of HRG-β1 (Fig. 1A). HRG-β1 caused scattering of MCF7 cells, which is consistent with previous results [27]. About 8 h after stimulation, the cell aggregates started to dissociate and this process continued for at least 24 h. (Fig. 1A).

### Association of F-actin with Adhesion Proteins is Lost Prior to Dissociation of the Cells

The localization of the actin cytoskeleton and the adhesion proteins, E-cadherin and occludin, were observed during dissociation of the cells. Before treatment with HRG-β1, colocalization of the actin cytoskeleton and the adherent proteins was detected (Fig. 1.B, C, D, and E). At 2 h after treatment with HRG-β1, F-actin colocalization with adherent proteins became faint and was completely lost at 8 h of treatment (Fig. 1.B, C, D, and E). Even though the actin cytoskeleton did not support the junctions at this stage of the treatment, the cells did not dissociate immediately, but remained associated for a few more hours. E-cadherin and occludin were still present at the cell–cell adhesion, suggesting that presence of adherent proteins is sufficient for cell–cell adhesion. A few hours later, the adherent junctions started to break down and cells underwent dissociation. How the junctions broke down is described in detail below.

**Figure 2:**
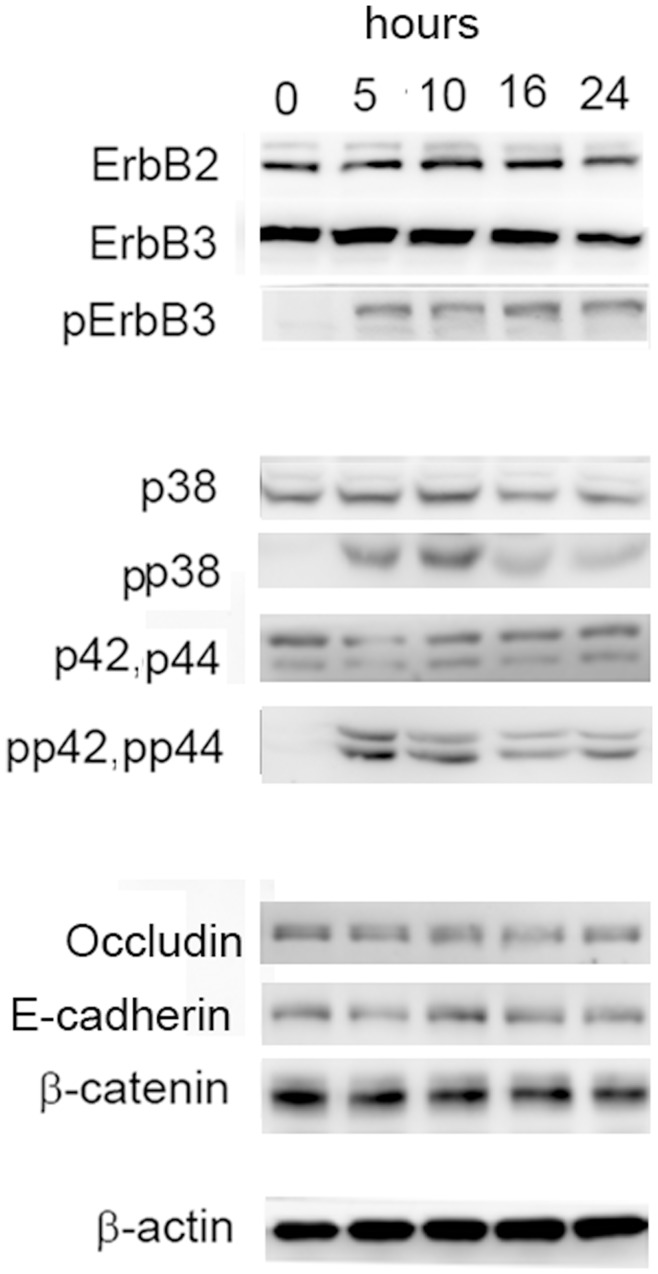
Protein levels during dissociation of the cells. MCF7 cells were treated with the EGF domain of HRG-β1 (100 ng/ml) for the indicated time. The indicated proteins were analyzed by Western blotting with the relevant antibodies.

During this procedure, phosphorylation of ErbB3 was still detectable at 24 h after HRG-β1 treatment, suggesting that the signal of HRG-β1 lasts for at least 24 h. (Fig. 2). The total amounts of ErbB3 and ErbB2, a partner of ErbB3, remained virtually unchanged (Fig. 2). The levels of occludin, E-cadherin, and β-catenin also remained unchanged even after the cells underwent dissociation (Fig. 2). These results suggest that signaling of HRG-β1 may act through the post-translational modification of proteins rather than by gene expression.

### MEK Inhibitors Block Dissociation of MCF7 Cells

**Figure 3:**
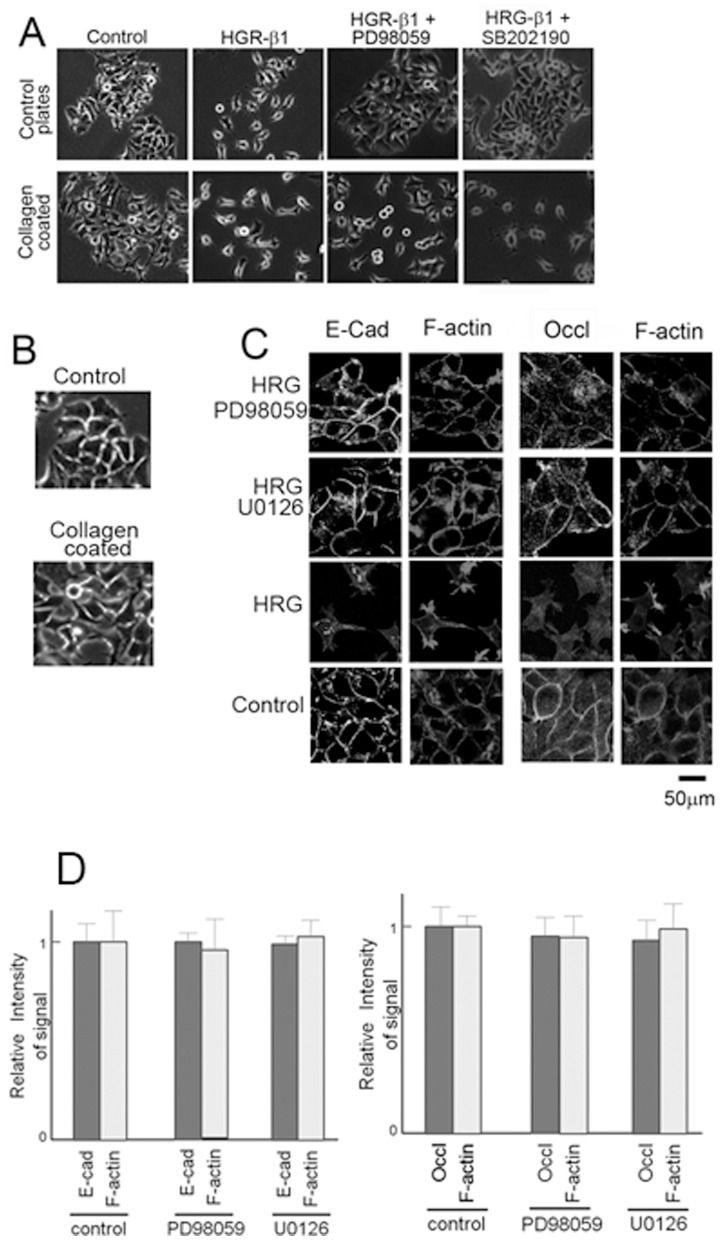
MEK inhibitors can block dissociation. A) Overnight cultures of MCF7 cells were treated with the EGF domain of HRG-β1 (100 ng/ml) for 24 h on plates coated with or without collagen. MEK inhibitor PD98059 (10 µM) was added to the cultures on some plates. As shown in the figure, PD98059 inhibited dissociation of MCF7 cells when collagen was absent. B) Enlarged images of the “control” of A. C) E-cadherin, occludin, and F-actin were stained. After inhibition of MEK, the disappearance of F-actin from cell–cell contact was blocked. F-actin co-localized with E-cadherin or occludin. D) Intensity of the signals of E-cadherin, occludin, and F-actin at the cell-cell contact was measured and expressed in the graph. Intensity of the control samples were expressed by 1.0. The error bars show standard deviation.

The MEK pathway, which is activated by the ErbB2/ErbB3 complex, has been shown to be important for various cell responses. Therefore, we explored the role of MEK in the dissociation of MCF7 cells. It has been suggested that MEK inhibitors do not affect the dissociation of MCF7 cells [27]. However, in our hands, the MEK inhibitors PD98059 and U0126 were capable of blocking dissociation of these cells (Fig. 3A). This difference was due to culture condition. The previous study was done on plates coated with collagen. Under this condition, MCF7 cells dissociated even when PD98059 was added to the medium. Treatment with U0126 gave a similar result (data not shown). MCF7 cells plated on a collagen plate formed loose colonies, whereas the colonies formed on the control plates were tight (Fig. 3B). It is likely that collagen contributes to MCF7 cell dissociation and that this activity makes the scattering of the cells resistant to MEK inhibition. The signaling pathways associated with the effects of collagen activity are totally unknown. The p38 MAP kinase inhibitor also failed to block scattering in collagen-coated plates (Fig. 3A). Thus, we used collagen-free dishes for the remainder of the study.

MCF7 cells were stimulated with HRG in the presence of the MEK inhibitors and the localization of F-actin, E-cadherin, and occludin were examined. F-actin (as well as E-cadherin and occludin) was found at the cell–cell contact of HRG-β1-treated cells (Fig. 3C and D), which suggests that the MEK activity is required for the disappearance of F-actin.

### Inhibition of p38 MAP Kinase Blocks Dissociation of MCF7 Cells

**Figure 4:**
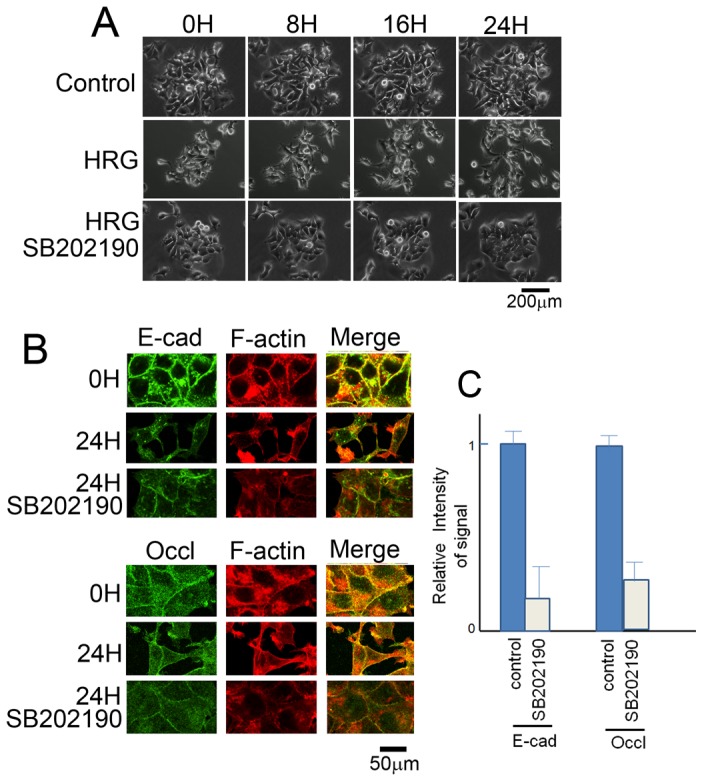
Effect of p38 MAP kinase inhibitor on dissociation of MCF7 cells. A) MCF7 cells were treated with the EGF domain of HRG-β1 (100 ng/ml) for 24 h in the presence or absence of SB202190 (1 µM). Time-lapse imaging of dissociation of the cells was performed. Although control cells moved, they never separated from the aggregate. In contrast, HRG-β1-treated cells underwent dissociation. At 8 h after HRG-β1 stimulation, cells started to dissociate. At 16 h after HRG-β1 stimulation, cells were rapidly dispersing. Presence of SB202190 inhibited dissociation as well as movement of cells, which produced very tight aggregates. B) MCF7 cells were treated with the EGF domain of HRG-β1 (100 ng/ml) for 24 h in the presence or absence of SB202190 (1 µM). Although E-cadherin and occludin were detected at the cell–cell contacts, their signals were much weaker or even absent, as compared to those in the aggregates formed without HRG-β1 treatment. F-actin was not detected at the cell–cell contacts. C) Brightness of E-cadherin and occludin at the junctions of control and HRG-β1-treated (24 h) cells in the presence of SB202190. Results are means of three independent experiments. Error bars show standard deviations. D) Intensity of the signals of E-cadherin, occludin, and F-actin was measured at the cell-cell contact and expressed in the graph. Intensity of the control samples were expressed by 1.0. The error bars show standard deviation.

Vadlamudi et al. found that scattering of MCF7 was dependent on the activity of p38 MAP kinase [27]. Indeed, p38 MAP kinase was activated during scattering of MCF7 cells treated with HRG-β1 (Fig. 2). Treatment of the cells with SB202190, an inhibitor of p38 MAP kinase, blocked the dissociation (Fig. 4A). Another p38 MAP kinase inhibitor, SB203580, produced similar results (data not shown). When dissociation of the cells was inhibited in the presence of HRG-β1, they appeared less spread out than the control cells (Fig. 4A). Therefore, we examined adhesion proteins and F-actin (Fig. 4B). E-cadherin and occludin were detected at the cell–cell contacts; however, the intensities of their signals were far fainter than those of the control cells (Fig. 4C and D). The signals of E-cadherin and occludin were occasionally discontinued, suggesting that the tight junctions and adherens junctions were not normal. Although the cells contained F-actin, it was not co-localized with E-cadherin or occludin (Fig. 4B). Thus, p38 MAP kinase-mediated dissociation of the cells may occur after the separation of F-actin from adherent proteins, which may be regulated by the MEK pathway.

### β-catenin is Released to the Cytosol from the Plasma Membrane Prior to other Adherent Proteins

**Figure 5:**
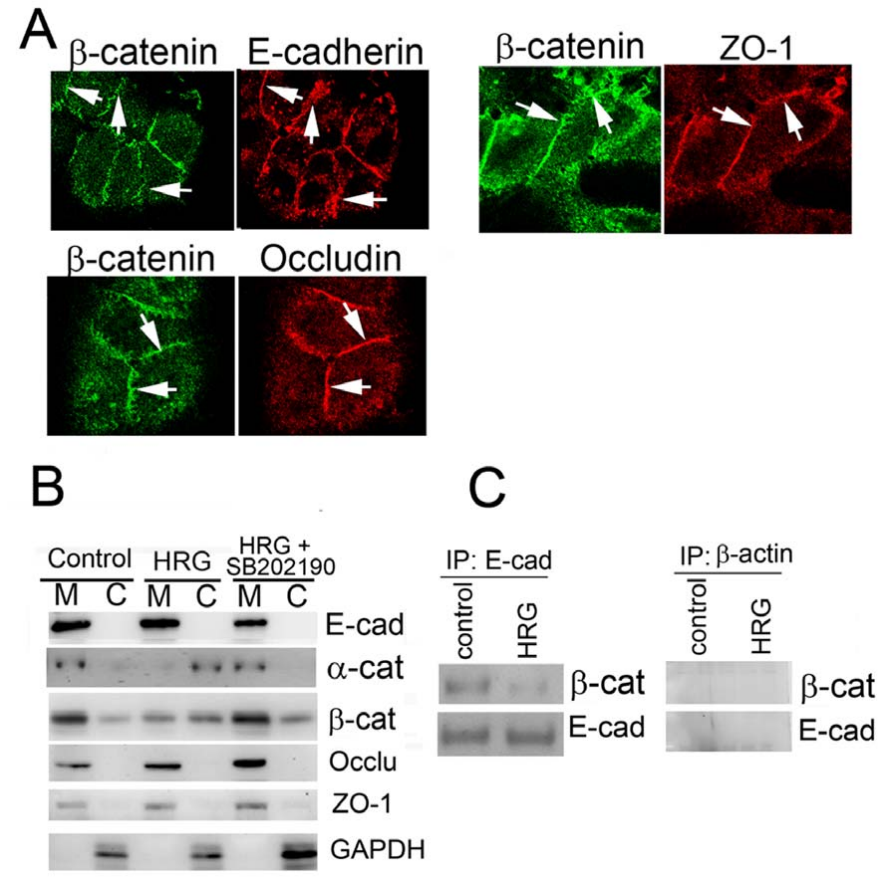
Release of adherent proteins from the junctions during dissociation. A) At 12 h after treatment with HRG-β1, cells were fixed and stained for the indicated proteins. The images are planes acquired from confocal microscopy. Of the four adherent proteins tested in this paper, the signals of β-catenin collapsed first. Arrows indicate where signals of E-cadherin, occludin, or ZO-1 were intact but those of β-catenin had collapsed. B) Cytoplasmic fractions of MCF7 cells treated with or without HRG-β1 in the presence or absence of SB202190 for 24 h were fractionated into membrane and the cytosol. E-cadherin and β-catenin were detected by Western blotting. GAPDH was used as a cytosol loading control. C) MCF7 cells were treated with or without HRG-β1 for 16 h. Then E-cadherin was immunoprecipitated. The samples were divided into two equal parts, and the levels of E-cadherin and co-immunoprecipitated β-catenin were examined by Western blotting with the relevant antibodies. The same experiment was done with anti-β-actin antibody as a negative control.

To understand how the junctions were broken down, we surveyed the adherent proteins while cells were dissociating. At the beginning, signals of E-cadherin, occludin, β-catenin, and ZO-1 were straight and clearly detected between neighboring cells. However, during dissociation of the cells, the signals became discontinued or zigzagged, which may reflect the release of the proteins from the adherent complexes. Using this approach, we tried to monitor the order of the release of proteins from the junctions. As shown in Fig. 5A, two adherent proteins were selected and stained with different colors. The shapes of the signals of the proteins were compared. In cases where the signal from one protein was straight and continuous while the signal for the other protein was zigzagged or fragmented, we interpreted this as the latter protein breaking down before the former protein. We repeated this experiment with different combinations to determine the temporal order of release of the adherent proteins from cell-cell junctions. The timing of the release of adherent proteins from cell–cell junctions was slightly different. At some point in the dissociation of cells, localization of β-catenin, which is strong and straight in HRG-β1 untreated cells, was discontinued or zigzagged while E-cadherin, occludin, and ZO-1 appeared to be intact. This suggests that β-catenin was released prior to the other proteins (Fig. 5A). However, observations using localization alone of adherent proteins is insufficient proof. Further study is required to definitively establish the order, if any, of the breakdown of these junctions. Still, these results implicate a temporal order in the release of adherent proteins from the plasma membrane upon HRG-β1 stimulation.

Dissociation of the adherent proteins was further examined biochemically. After treatment with HRG-β1 for 24 h, cells were fractionated, and the cytoplasmic levels of E-cadherin and β-catenin were examined. As shown in Fig. 5B, E-cadherin was always detected in the membrane fraction, because it is a membrane-associated protein that binds to the inner membrane when internalized. In contrast, β-catenin was found in the membrane and cytosolic fractions. Because β-catenin transduces Wnt signaling in addition to HRG-β1 signaling, the presence of β-catenin in the cytosol is expected. After treatment with HRG-β1, membrane-bound β-catenin decreased and cytosolic β-catenin increased, reaching a ratio of about 1∶1 (compared to about 4∶1 before stimulation), suggesting that the adherens junction complex broke down upon internalization. Presence of SB202190 maintained the ratio of β-catenin in membrane and cytosol to 4∶1, suggesting that the adherent complex was not broken down when dissociation of the cells was inhibited by this drug (Fig. 5B). We also examined the localization of α-catenin. As shown in Fig. 5B, α-catenin also translocated to the cytosol. Therefore, α-catenin and β-catenin are the adherent proteins that moved from the membrane fraction to the cytosol fraction during cell dissociation. Occludin and ZO-1 stayed in the membrane fraction (Fig. 5B). To confirm this data, co-immunoprecipitation of E-cadherin and β-catenin was examined. As shown in Fig. 5C, β-catenin binding to E-cadherin dramatically decreased. These results support the idea that the adherent complex is absent at the plasma membrane when cells dissociate.

### Inhibition of p38 MAP Kinase Induces Re-aggregation of MCF7 Cells Already Dissociated by HRG-β1 Treatment

**Figure 6:**
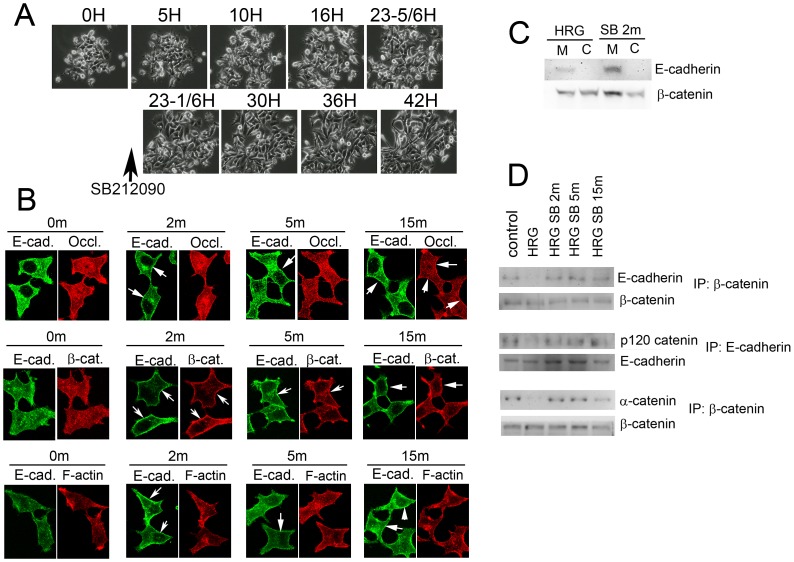
Localization of E-cadherin, occludin, and β-catenin in re-aggregating cells after treatment with SB202190. A) MCF7 cells were treated with HRG-β1 for 24 h to allow dissociation. Then SB202190 and another dose of HRG-β1 were added. Cells were monitored by time-lapse imaging. Cells were dissociated, but after addition of SB202190 they started to re-aggregate within 10 min. B) MCF7 cells were treated with the EGF domain of HRG-β1 (100 ng/ml) for 22 h. Then SB202190 was added to the culture and incubated for the indicated time. After fixation, the indicated proteins were stained and observed by confocal microscopy. Images are the sections at around the middle of the cells. Arrows indicate where the proteins are accumulated at the plasma membrane. E-cadherin and β-catenin moved to the plasma membrane within 2 min after stimulation with HRG-β1, whereas occludin did not move to the plasma membrane until 15 min after stimulation. Although E-cadherin and β-catenin accumulated at the plasma membrane, some signal later returned to the cytosol. F-actin did not move to the plasma membrane. C) MCF7 cells were stimulated with HRG-β1 for 20 h and SB202190 was added to the medium for 2 min. The reaction was stopped by addition of a low-salt buffer and cellular fractionation was performed. E-cadherin and β-catenin were detected by Western blotting. HRG: before addition of SB202190; SB 2 m: incubation with SB202190 for 2 min. D) MCF7 cells were treated with HRG-β1 for 16 h. Then the cells were treated with SB202190 for the indicated time. Cells were lysed and the immunoprecipitation was performed using the indicated antibodies. The samples were divided into two equal parts and the levels of the indicated proteins in the immunoprecipitates were detected by Western blotting with the relevant antibodies.

The effect of SB202190 on the cells that were dissociated after HRG-β1 treatment was examined. As shown in Fig. 6A, the cells immediately started to re-aggregate. This result suggests that continuous activation of p38 MAP kinase is required for maintenance of the dissociated phenotype. Even in the presence of HRG-β1, the cells continued to aggregate and form considerable sized colonies.

The mechanism of re-aggregation was examined by monitoring adherent proteins. After addition of SB202190, the proteins of the adherens junction complex, which were diffusely distributed inside the cell, rapidly moved to the plasma membrane (Fig. 6B). Within 2 min of treatment E-cadherin and β-catenin translocated to the plasma membrane concurrently, suggesting that they bind together before moving to the plasma membrane. Later, occludin translocated to the plasma membrane (Fig. 6B). This observation was further confirmed through cell fractionation studies as above. As shown in Fig. 6C, membrane-bound β-catenin levels, which were equal to cytosolic β-catenin levels in HRG-β1-treated cells, were highly enriched after incubation with SB202190 for 2 min, confirming that β-catenin moved to the plasma membrane after inhibition of p38 MAP kinase activity. The formation of adherens junction complex was also tested. As shown in Fig. 6D, the binding of β-catenin to E-cadherin increased dramatically within 2 min, which coincided with the translocation of these proteins to the plasma membrane. Other components of the adherens junction complex were also monitored. As shown in Fig. 6E, binding of p120 catenin to E-cadherin and α-catenin to β-catenin were readily detectable in control cells and highly diminished in dissociated cells. Upon treatment with SB202190, these interactions were restored within 2 min, suggesting that all the components of the adherens junction complex separated during dissociation and reassembled when the cells formed aggregates. F-actin did not move to the plasma membrane, suggesting that it is not regulated by the p38 MAP kinase pathway.

Next, cells were immunostained for E-cadherin, occludin, and F-actin. The signals for E-cadherin and occludin were readily detectable at the cell–cell contact, although intensity of the signals was weak compared to the control. However, association of F-actin with these adhesion proteins was not detected. These results suggest that actin is not required to support cell–cell adhesion. However, the junctions were rigid and little movement of the cells was observed (data not shown). Thus, F-actin is probably required for flexibility of the junctions. This pattern was very similar to the one seen when dissociation was inhibited by the p38 MAP kinase inhibitors (see Fig. 4B).

## Discussion

### The p38 MAP Kinase and the MEK Pathway Regulate Dissociation of MCF7 Cells

In this study, we showed that HRG-β1 regulates cell–cell adhesion via the p38 MAP kinase cascade. Dissociation of the cells after treatment with HRG-β1 was reported in previously [27]. However, the study was limited to only a short time after treatment with HRG-β1, and how MCF7 cells dissociate was not examined. Instead, the paper reports the presence of membrane ruffling a short time after HRG-β1 treatment. Membrane ruffling was also dependent on p38 MAP kinase. Therefore, p38 MAP kinase is responsible for early and late events in dissociation, as described in our study. Here, we also demonstrated that p38 MAP kinase regulates adhesion proteins such as E-cadherin and occludin.

As mentioned above, p38 MAP kinase functions at various points during dissociation of MCF7 cells. Indeed, phosphorylation of p38 MAP kinase was observed throughout the dissociation process, which may be associated with the prolonged activation of ErbB3 after HRG β-1 stimulation. During cell dissociation, adherens and tight junctions are lost. The F-actin backbone disappears first, followed by E-cadherin and occludin from the cell–cell contacts. MEK activity is required for F-actin breakdown. When MEK is inhibited, tight and adherens junctions remain intact and p38 MAP kinase-mediated dissociation of the cells is not observed. Regulation of F-actin was independent of cell–cell adhesion and not required for cell–cell adhesion. The MEK pathway was important for the regulation of F-actin but not for cell–cell adhesion.

### Dynamics of Adherent Proteins

For breakdown of cell–cell interaction, it appears that adherens junctions are lost prior to tight junctions. Among the four proteins tested in this paper, β-catenin may be the first to be released from the cell–cell contacts. These results suggest that p38 MAP kinase signaling triggers the breakdown of adherens junctions on the plasma membrane. This leads to the depletion of tight junctions.

When p38 MAP kinase signaling was interrupted in HRG β1-dissociated cells, cytosolic E-cadherin and β-catenin immediately moved to the plasma membrane. This reaction was very rapid (within 2 min of treatment), suggesting that the p38 MAP kinase pathway may exert its affect through post-translational modification of adherent proteins. Indeed, 10 min after addition of SB202190, association of the cells had already started (Fig. 6A). Given that adherens junction complexes break down sequentially during dissociation and E-cadherin and β-catenin translocate to the plasma membrane simultaneously during association, it is likely that p38 MAP kinase signaling regulates adherens junctions at the level of the plasma membrane. Once p38 MAP kinase signaling is turned off, adherent proteins translocate to the plasma membrane where they presumably reassemble. Since adherens junction proteins move to the plasma membrane before occludin, p38 MAP kinase signaling reaches adherens junction proteins prior to tight junction proteins. Therefore, the regulation of cell–cell contact from inside of the cells may be dependent on adherens junction.

### Epithelial Cells may have an Intrinsic Ability to Bind to Neighboring Cells

Dissociation is associated with the loss of cell–cell interaction, which is a characteristic of poorly differentiated adenocarcinomas. To maintain the dissociated state, continuous activation of p38 MAP kinase is required. When the signal from p38 MAP kinase is blocked, cells immediately regain the ability to bind to neighboring cells. These findings suggest that epithelial cells have an intrinsic capacity to bind to neighboring cells unless a specific signal for dissociation from neighboring cells is present. It is likely that cell dissociation *in vitro* is an artificial phenomenon caused by continuous treatment of the cells with growth factors, which is not the case *in vivo*. Instead, this reversible pathway seems to control cell–cell dissociation for only short periods such as cell division. Cells restrict the dissociation period for special occasions *in vivo*. Therefore, disorder in the mechanism of dissociation may be related to the formation of malignant and poorly differentiated adenocarcinomas. As shown in this paper, activities of adherent proteins are important for the regulation of cell–cell interaction. Understanding how these activities are regulated may contribute to the therapy of malignant tumors.
